# Lactic Acid-Producing Probiotic *Saccharomyces cerevisiae* Attenuates Ulcerative Colitis *via* Suppressing Macrophage Pyroptosis and Modulating Gut Microbiota

**DOI:** 10.3389/fimmu.2021.777665

**Published:** 2021-11-24

**Authors:** Siyuan Sun, Xiuxiu Xu, Ling Liang, Xiaoli Wang, Xue Bai, Lanping Zhu, Qijin He, Huixi Liang, Xin Xin, Li Wang, Chenxi Lou, Xiaocang Cao, Xin Chen, Bingzhi Li, Bangmao Wang, Jingwen Zhao

**Affiliations:** ^1^ Department of Gastroenterology and Hepatology, Tianjin Medical University General Hospital, Tianjin Institute of Digestive Disease, Tianjin Key Laboratory of Digestive Diseases, Tianjin, China; ^2^ Frontier Science Center for Synthetic Biology and Key Laboratory of Systems Bioengineering (Ministry of Education), School of Chemical Engineering and Technology, Tianjin University, Tianjin, China

**Keywords:** ulcerative colitis, lactic acid, *Saccharomyces cerevisiae*, pyroptosis, gut microbiota

## Abstract

Lactic acid, a metabolic by-product of host and intestinal microbiota, has been recovered as an active signal molecule in the immune system. In this study, a lactic acid biosynthesis pathway that directly produces lactic acid from glucose rather than ethanol with high production was reconstructed in *Saccharomyces cerevisiae*. The engineered *S. cerevisiae* showed anti-inflammatory activity in dextran sulfate sodium (DSS)-induced mice with improved histological damage, increased mucosal barrier, and decreased intestinal immune response. Lactic acid regulated the macrophage polarization state and inhibited the expression of pro-inflammatory cytokines *in vivo* and *in vitro*. Increasing the macrophage monocarboxylic acid transporter-mediated active lactic acid uptake suppressed the excessive activation of the NLRP3 inflammasome and the downstream caspase-1 pathway in macrophages. Moreover, lactic acid promoted histone H3K9 acetylation and histone H3K18 lactylation. Meanwhile, the engineered *S. cerevisiae* altered the diversity and composition of the intestinal microbiota and changed the abundance of metabolic products in mice with colitis. In conclusion, this study shows that the application of engineered *S. cerevisiae* attenuated DSS-induced colitis in mice *via* suppressing macrophage pyroptosis and modulating the intestinal microbiota, which is an effective and safe treatment strategy for ulcerative colitis.

## Introduction

Inflammatory bowel diseases (IBDs) are chronic idiopathic nonspecific and relapsing gastrointestinal diseases that are mainly categorized into two types: Crohn’s disease and ulcerative colitis (UC) (1). UC is limited to the colon and is characterized by weight loss, diarrhea, and abdominal pain and affects people of all ages. Although the etiology and pathogenesis remain unclear, it is widely believed to be the result of genetics and immunological, microbial, and environmental factors (2–4).

Macrophages, as mature forms of monocytes, participate in both innate and adaptive immunity. They usually are the first defense in innate immunity (5). Once pathogens break through the epithelial barrier and invade the intestinal mucosa, they can be recognized by macrophages with Toll-like receptors, pattern recognition receptors (PRRs), and others, which further induce a series of pathway responses such as the NLRP3 inflammasome and finally cause cell membrane rupture with the cytokines interleukin 1β (IL-1β) and IL-18, with the cell contents released. This type of inflammatory cell death is termed pyroptosis (6, 7). Like other innate immune responses, pyroptosis is beneficial for host’s self-defense against bacterial, fungal, and viral infections (8). However, a dysregulated pyroptosis intimately contributes to the development of UC *via* disrupting the intestinal epithelial barrier and inducing the dysregulation of adaptive immunity by promoting Th17 cells to produce IL-17 and Th1 cells to produce interferon gamma (IFN-γ) (9, 10). It has been proven that suppressing the pyroptosis of macrophages may be a novel strategy to cure experimental colitis (11, 12).

Immune cells can be metabolically reprogrammed to modulate their functions. Lactic acid, being a metabolic substrate, has been re-recognized as an active signal in the functions of regulatory immune cells (13, 14). Previous studies have revealed various mechanisms of how immune cells in diseased tissues respond to the local accumulation of metabolites. Lactic acid mostly exists in the form of ions in the gut, which is mainly absorbed and utilized by the monocarboxylic acid transporter 1 (MCT1) (15). Moreover, lactic acid downregulated cyclic AMP (cAMP) and protein kinase A (PKA) signaling *via* binding to the GPR81 receptor on the surface of intestinal macrophages, further inhibiting the expression of pro-inflammatory factors (16). Lactic acid could protect the heart and ischemic neurons, promote adult hippocampal neurogenesis, and inhibit inflammation following organ injury (17). Lactic acid also promotes histone H3 lysine lactylation in macrophages and alters the macrophage polarization state by increasing the expressions of *Arg-1* and other M2-like macrophage genes (18). However, whether lactic acid modulates the immune responses in colitis remains unclear.

Nowadays, live biotherapeutic products (LBPs) are emerging as effective treatments for inflammatory diseases *via* improving nutrient absorption and the host defense system (19). *Saccharomyces cerevisiae* is a facultative anaerobic fungus that has been widely used to develop oral vaccines and engineered carriers in medicine (20, 21). More studies have made it an engineering vector and transformed it to highly express small-molecule drugs (22, 23). The non-pathogenic *S. cerevisiae* has not been widely used as a probiotic. Its beneficial properties have been demonstrated in the treatment of a variety of diseases, including improving the gut immune response and the intestinal barrier. *Saccharomyces boulardii* supplementation may improve the therapeutic effect in the treatment of IBDs (24). *S. cerevisiae*, as a healthy microorganism, is reduced in IBD and has been associated with the physiological response of the immune system. In fact, based on its ability to increase the level of IL-10, supplementation with *S. cerevisiae* has been proposed as an anti-inflammatory method. Recent advances in synthetic biology have enabled probiotic engineering to provide therapeutic components in response to disease-related signaling pathways. Constructing *S. cerevisiae via* synthetic biology methods makes up for the defects of the natural strain and has the functional characteristics of acid resistance, easy colonization, and high-efficiency expression. Recently, the developed self-tunable engineered yeast that produces apyrase in response to extracellular ATP (eATP) levels has shown a dynamic anti-inflammatory effect on intestinal inflammation in IBD (25).

Herein, we used *S. cerevisiae* as a chassis cell to improve the production of lactic acid *via* synthetic biology methods and demonstrated the protective effect of engineered *S. cerevisiae* on mice with colitis. Moreover, we further explored the underlying mechanism of lactic acid inhibiting the pyroptosis of macrophages. This work will lay the foundation for the clinical development of IBD treatment based on the optimized engineering strain for probiotic yeast transplantation.

## Materials and Methods

### Reagents and Antibodies

The different reagents used were l-(+)-lactic acid (L6402), lipopolysaccharide (LPS) from *Escherichia coli* O111:B4 (L2630), polystyrene latex beads (2.0 μm, L4530), and nigericin (481990), which were purchased from Sigma-Aldrich (St. Louis, MO, USA). The antibodies for Western blot against NLRP3 (NBP-12446) and caspase-1 (14F468) were purchased from Novus (Littleton, CO, USA). Antibodies against gasdermin-D (97558S), IL-1β (12242S), and histone H3 (4499S) were purchased from Cell Signalling Technology (CST; Danvers, MA, USA). Antibodies against histone H3 (acetyl K18, ab40888; acetyl K9, ab32129) and histone H4 (acetyl K5, ab51997) were purchased from Abcam (Cambridge, UK). Antibodies against histone lactyllysine (PTM-1401) and histone H3 (lactyl K9, PTM-1419RM; lactyl K18, PTM-1406) were purchased from PTM BIO (Zhejiang, China). The antibodies for immunohistochemistry against MUC2 (GB111965) was purchased from Servicebio (Wuhan, China). The antibodies for immunofluorescence against zonula occludens-1 (ZO-1, 33-9100) were purchased from Invitrogen (Carlsbad, CA, USA) and those for immunofluorescence against F4/80 (70076) and CD206 (AF2534) were from CST and R&D System (Minneapolis, MN, USA), respectively. The antibodies for flow cytometry, including phycoerythrin (PE) anti-mouse F4/80 (123110), fluorescein isothiocyanate (FITC) anti-mouse/human CD11b (101206), allophycocyanin (APC) anti-mouse CD86 (105012), and APC anti-mouse CD206 (141708) were purchased from BioLegend (San Diego, CA, USA). The stool hemoccult kit (C027-1-1) was purchased from Nanjing Jiancheng Bioengineering Institute (Nanjing, China) and the lactic acid assay kit II (K627-100) purchased from Solarbio (Beijing, China). Cytochalasin D (ab141788) was purchased from Abcam, and the MCT1 inhibitor AZD3965 (HY1250) was purchased from MedChemExpress (Princeton, NJ, USA). Macrophage colony stimulating factor (M-CSF; 416-ML) was purchased from R&D Systems. Information on the sequences used for strains is listed in **Table 1**.

**Table 1:** Primers used in this study.

Gene	Sequence (5′→3′)
Mice-GAPDH	Forward: GGAGAAACCTGCCAAGTATG Reverse: TGGGAGTTGCTGTTGAAGTC
Mice-IL-6	Forward: CCAGTTGCCTTCTTGGGACT Reverse: GGTCTGTTGGGAGTGGTATCC
Mice-iNOS	Forward: GAGACAGGGAAGTCTGAAGCAC Reverse: CCAGCAGTAGTTGCTCCTCTTC
Promoter-PDC5	Forward: CCCGCGGCCGCCAACAAAGGCCAAGGAAATAAAGCA Reverse: CATGAGTTTTATGTTAATTAGCTTATTTGTTCTTCT TGTTATTGTATTGTGTTGTTC
Terminator-PDC5	Forward: GAACAACACAATACAATAACAAGAAGAACAAATAA GCTAATTAACATAAAACTCAT Reverse: CTTCAATTTAATTATATCAGTTATTACCCTGTAGTATTC GAGCATTAAGGA
Ura3-PDC5	Forward: TCCTTAATGCTCGAATACTACAGGGTAATAACTGATAT AATTAAATTGAAG Reverse: TACCTAAGGTTATTTCAGACATGATTCGGTAATCTCCG AACAG
Part of the ORF-PDC5	Forward: CTGTTCGGAGATTACCGAATCATGTCTGAAATAACCT TAGGTA Reverse: GGGCGGCCGCCGTTAGCAATATCAGTGATCA
Promoter-PDC6	Forward: CCCGCGGCCGCGAGTTCACACCTTATTCACA Reverse: GTTTGAGTACACTACTAATGGCTTATTTGTTGGCA ATATGTTTTTGCTATATTACGTG
Terminator-PDC6	Forward: CACGTAATATAGCAAAAACATATTGCCAACAAATAAGCC ATTAGTAGTGTACTCAAAC Reverse: CTTCAATTTAATTATATCAGTTATTACCCATGCAGAATGA GCACTTGTTA
Ura3-PDC6	Forward: TAACAAGTGCTCATTCTGCATGGGTAATAACTGATAT AATTAAATTGAAG Reverse: TCCAAGAGTAATTTCAGACATGATTCGGTAATCTCCG AACAGAAGGAA
Part of the ORF-PDC6	Forward: CTTCTGTTCGGAGATTACCGAATCATGTCTGAAATTAC TCTTGGA Reverse: GGGGCGGCCGCGTTGTCCTGATCAACCTATC
Promoter-ADH4	Forward: CCCGCGGCCGCGCCTTCAATGAGCTTCCAT Reverse: TTGACGTTTATGAGTTCGTTCGATTTTTTTATTTTCTTAT TTGACTATTAGTTGTTGCA
Terminator-ADH4	Forward: TGCAACAACTAATAGTCAAATAAGAAAATAAAAAAATC GAACGAACTCATAAACGTCAA Reverse: CTTCAATTTAATTATATCAGTTATTACCCAAGGAAGACA ATATCATTGACAG
Ura3-ADH4	Forward: TGTCAATGATATTGTCTTCCTTGGGTAATAACTGATATA ATTAAATTGAAG Reverse: AAACCCAGTAACGGAAGACATGATTCGGTAATCTCCG AACAGAAGGAA
Part of the ORF-ADH4	Forward: TTCTGTTCGGAGATTACCGAATCATGTCTTCCGTTACTG GGTTT Reverse: GGGGCGGCCGCTGGCAAACCAAACATGGTAGA
Promoter-PDC1	Forward: CCCGCGGCCGCCTGACTTTTCGTGTGATGAGG Reverse: GATCAATTGGTCCTTCAAAGTAGCCATTTTGATTG ATTTGACTGTGT
Terminator-PDC1	Forward: TCCAAAAGGAATTGCAATTCTAAGCGATTTAATCTC TAATTATTAGTTAAAGT Reverse: CTTCAATTTAATTATATCAGTTATTACCCACAACTAGCTTGT CTTGAGCA
Ura3-PDC1	Forward: TGCTCAAGACAAGCTAGTTGTGGGTAATAACTGATATAA TTAAATTGAAG Reverse: TTACCCAAAGTAATTTCAGACATGATTCGGTAATCTCCGA ACAGA
Part of the ORF-PDC1	Forward: TCTGTTCGGAGATTACCGAATCATGTCTGAAATTACTTTGG GTAA Reverse: GGGGCGGCCGCTAAGTGGTTCTGATACATCTGTCA
LDH-ΔPDC1∷LDH	Forward-ACACAGTCAAATCAATCAAAATGGCTACTTTGAAGGA CCAATTGATC Reverse: ACTTTAACTAATAATTAGAGATTAAATCGCTTAGAATTG CAATTCCTTTTGGA
Promoter-ADH1	Forward: CCCGCGGCCGCTCCATTTGCCATCTATTGAAGT Reverse: TTGGTCCTTCAAAGTAGCCATTGTATATGAGATAGTT GATTGT
Terminator-ADH1	Forward: TCCAAAAGGAATTGCAATTCTAAGTTGACACTTCTA AATAAGCGAA Reverse: CTTCAATTTAATTATATCAGTTATTACCCAACGTCTTGAAG CTCAGGTAAG
Ura3-ADH1	Forward: CTTACCTGAGCTTCAAGACGTTGGGTAATAACTGATATAAT TAAATTGAAG Reverse: ACACCTTTTTGAGTTTCTGGGATAGACATGATTCGGTAAT CTCCGAACAGA
Part of the ORF-ADH1	Forward: TCTGTTCGGAGATTACCGAATCATGTCTATCCCAGAAACT CAAAAAGGTGT Reverse: GGGGCGGCCGCGTGATACCAGCACACAAGATG
LDH-ΔADH1∷LDH	Forward: ACAATCAACTATCTCATATACAATGGCTACTTTGAAGG ACCAA Reverse: TTCGCTTATTTAGAAGTGTCAACTTAGAATTGCAATT CCTTTTGGA

### Saccharomyces cerevisiae

#### Strains

*S. cerevisiae* BY4741 (MATa, his3, leu2, met15, and ura3) was used for the construction of the recombinant strain. The gene-deleting modules were constructed to knock out the genes *PDC1*, *PDC5*, *PDC6*, *ADH1*, and *ADH4*, and the strain obtained was marked as *S.cerevisiae* 80#. Then, the exogenous gene *LDH* was inserted at the *PDC1* and *ADH1* knockout positions at the same time in order to obtain the strain marked as *S.cerevisiae* 39#. The primers used for the construction of the gene-deleting modules are shown in **Table 1**.

### Media and Culture Condition

*S. cerevisiae* strain BY4741 was cultivated in liquid yeast extract peptone dextrose (YPD) medium containing 20 g/L glucose, 10 g/L yeast extract, and 20 g/L peptone. The recombinant strains with the *Ura3* gene were cultivated in liquid SC-Ura medium (synthetic complete medium without uracil; 6.7 g/L yeast nitrogen base without amino acids, 20 g/L glucose, 0.1 g/L leucine, 0.02 g/L histidine, and 0.02 g/L tryptophan). The recombinant strains without the *Ura3* gene were cultivated in liquid SC-5-FoA medium (synthetic complete medium with 5-FoA; 1 g/L 5-FoA, 6.7 g/L yeast nitrogen base without amino acids, 20 g/L glucose, 0.1 g/L leucine, 0.02 g/L histidine, 0.02 g/L tryptophan, and 0.5 g/L uracil). The growth of the yeast strains during cultivation were measured at a wavelength of optical density (OD) 600 nm using a spectrophotometer. The lactic acid concentrations were measured using the LA Assay Kit (BC2235, Solarbio).

### Animal Experiments

Twenty-six male C57BL/6 mice (6–8 weeks old; specific pathogen free, SPF) were purchased from Hua Fu Kang (Beijing, China). Dextran sulfate sodium (DSS, molecular weight = 36–50 kDa) (MP Biomedicals, Santa Ana, CA, USA) was used in this experiment. The mice were housed in a SPF facility at the Laboratory Animal Center of the Chinese Academy of Medical Sciences, Institute of Radiation Medicine. The mice were randomly assigned into four groups: Water+PBS, DSS+PBS, DSS+LAC, and DSS+SyBE. After 1 week of accommodation, six mice per cage were housed at room temperature (standard light cycle, 12/12-h light/dark) with free access to food and water. Treatments were maintained for 7 days. The mice in the DSS+PBS, DSS+LAC, and DSS+SyBE groups received 2% (*w*/*v*) DSS in drinking water, while those in the Water+PBS group received normal drinking water. Meanwhile, the mice in the Water+PBS and DSS+PBS groups received phosphate-buffered saline (PBS, 0.2 ml) per day by intragastric gavage, those in the DSS+LAC group received 0.25 mM lactic acid (0.2 ml/day) by intragastric gavage, and the mice in the DSS+SyBE group received 10^9^ CFU/ml *S.cerevisiae* 39# strain. The body weights and feces of the mice were recorded daily, and their disease activity index (DAI) was scored according to **Table 2**. Feces of mice were collected in sterile Eppendorf tubes and stored at −80°C. On the last day, the mice were sacrificed by cervical dislocation under anesthesia, the blood was collected, the colons were removed and the lengths measured, and the tissues were fixed in 4% paraformaldehyde solution or stored at −80°C until further analysis. All animal welfare and experimental procedures complied with the Laboratory Animal Management Regulations in China and the related ethical regulations of Tianjin Medical University.

**Table 2:** Disease activity index (DAI) scoring system.

Score	Weight loss (%)	Stool consistency	Occult/gross bleeding
0	None	Normal	Normal
1	1–5	Normal	Normal
2	5–10	Loose stool	Hemoccult positive
3	10–20	Loose stool	Hemoccult positive
4	>20	Diarrhoea	Gross bleeding

Scores were tallied for each category and then divided by 3 to obtain the DAI.

### Microphage Isolation and Treatment

To obtain bone marrow-derived macrophage (BMDM), 3- to 4-week-old healthy mice were sacrificed and the femurs were removed. The bone marrows were flushed with 5 ml Dulbecco’s modified Eagle’s medium (DMEM) containing 5% fetal bovine serum (FBS) with 1% penicillin–streptomycin. Then, the cell suspension was centrifuged and extracted with 60% and 30% Percoll. The cells were then plated onto 10-cm Petri dishes in DMEM containing 20% FBS, 1% penicillin–streptomycin, and 50 ng/ml M-CSF. The culture medium was replenished every 2 days. After 7 days, the cells were collected for analysis. To induce NLRP3 activation, BMDMs were plated at a density of 1.5 × 10^6^ cells/well in a 60-mm dish for 24 h and treated with 500 ng/ml LPS for 6 h with or without 15 mM lactic acid. Then, the cells were collected and total proteins were extracted. To determine macrophage polarization and phagocytosis, BMDMs were treated with 100 ng/ml LPS for 24 h with or without 15 mM lactic acid.

### Histological Analysis and Immunohistochemistry

The colon tissues fixed in 4% paraformaldehyde solution were paraffinized and sectioned (5 μm thick). Then, the sections were stained with hematoxylin and eosin (H&E). For immunohisto-chemistry, the sections were deparaffinized and rehydrated, processed with microwave antigen retrieval, and incubated overnight with primary antibodies against MUC-2 at 4°C. They were further incubated with secondary antibodies and streptavidin–horseradish peroxidase with diaminobenzidine.

### Immunofluorescent Staining

The colon sections were fixed in 100% acetone for 5 min at −20°C and then incubated with primary antibodies against F4/80, CD206, and ZO-1 overnight at 4°C. Then, the sections were incubated with appropriate secondary antibodies for 60 min. The sections were then incubated with an antifade mounting medium (with DAPI) for nuclear counterstaining. Images were obtained with a Leica fluorescence microscope (Leica, Wetzlar, Germany).

### Cytokine Measurement

For the quantification of cytokines in the serum, bead-based LEGENDplex™ analysis (BioLegend, San Diego, CA, USA) was used according to the manufacturer’s protocol. Six related cytokines were selected: IL-1β, IL-6, IL-10, IL-12p70, IFN-γ, and tumor necrosis factor alpha (TNF-α). Analysis was performed with the BD FACSCanto II flow cytometer (Becton Dickinson, San Diego, CA, USA), expressed in nanograms per milliliter.

### Western Blot Analysis

Basically, samples of colon were homogenized in ice-cold RIPA lysis buffer containing 1% protease inhibitor cocktail and centrifuged at 13,000 rpm for 15 min at 4°C. To extract histone, the cells were cultured in a 10-cm plate and homogenized in 0.5 ml lysis buffer containing 8 M urea, 1% phenylmethylsulfonyl fluoride (PMSF), 3 μM trichostatin, and 50 mM niacinamide. Then, the cell homogenate was ultrasonically split under conditions of 200 W, intermittent ratio 30:30 s/s, 4°C, for 20 min using an ultrasonic homogenezier (Scientz-IID, Xinzhi, China). The protein concentration was quantified using a bicinchoninic acid (BCA) protein assay kit. Fifty micrograms of the protein was loaded and separated in sodium dodecyl sulfate (SDS) polyacrylamide gels and transferred into 0.22-μm nitrocellulose filter membranes using wet transfer under conditions of 90 V, 90 min. The histones were transferred using the semi-dry transfer method (Bio-Rad, Hercules, CA, USA) under conditions of 20 V, 20 min. Then, the membranes were blocked with 5% (*w*/*v*) non-fat milk in 0.05% Tris-buffered saline (TBS) for 90 min at room temperature and then incubated with the primary antibodies overnight at 4°C. After being washed with TBST (TBS with Tween 20), the membranes were incubated with the respective secondary antibodies, including goat anti-mouse IgG (1:5,000, Zhong Shan Jin Qiao) and goat anti-rabbit IgG (1:5,000, Zhong Shan Jin Qiao), for 60 min at room temperature. Finally, the bands were visualized with enhanced chemiluminescence. The densitometry of the immunoblots was quantified using ImageJ software. β-actin was adopted as the internal standard to control the variation, and the relative protein expression values were expressed as fold mead of the controls compared to the corresponding control value. The control value was normalized to 1.0.

### RT-PCR

Total RNA was extracted from the colon tissues or cells using the Trizol reagent, and 2,000 ng RNA was reverse transcribed into complementary DNA (cDNA) using a cDNA systhesis kit (TIANGEN, Beijing, China). Quantitative real-time polymerase chain reaction (RT-PCR) was performed with the SYBR Green Master Mix (Applied Biosystems, Waltham, MA, USA). Relative messenger RNA (mRNA) expression was calculated using 2^−ΔΔ^ *^C^* ^t^. Glyceraldehyde-3-phosphate dehydrogenase (GAPDH) was used as the reference gene. The primer pairs used are presented in **Table 1**.

### Flow Cytometry Analysis

All data acquisition was performed using a Beckman cytometer. The CytExpert software was used for data analysis and graphical representation. Basically, BMDMs were stimulated with PBS and LPS (100 ng/ml) with or without lactic acid (15 mM) for 24 h. For fluorescence-activated cell sorting (FACS) analysis, BMDMs were collected and stained with the manufacturer’s suggested concentrations of FITC anti-CD11b, PE anti-F4/80, and APC anti-CD86 for 15 min at room temperature in the dark. For intracellular staining, the cells were stained with FITC anti-CD11b and PE anti-F4/80, fixed in a fixation buffer (BioLegend) for 20 min, and resuspendend with intracellular staining permeabilizaiton buffer. Finally, the cells were incubated with APC anti-CD206 and quantified.

### Phagocytosis Analysis

Carboxylate-modified polystyrene latex beads were pre-incubated in PBS with 10% FBS for 1 h at 37°C. The coated beads were added into LPS- or lactic acid-pretreated BMDMs at a ratio of 10:1. After phagocytosis for 2 h, non-adherent beads were washed twice with cold PBS. The cells were further detached by scraping with a soft rubber policeman and detected with a flow cytometer.

### Microbiota Analysis

Fecal samples were snap frozen in liquid nitrogen and stored at −80°C after collection. The DNA of total bacteria was extracted from the fecal samples using a QIAamp^®^ Fast DNA Stool Mini Kit. For 16S ribosomal RNA (rRNA) gene sequencing, the DNA samples were sent to the Center for Genetic & Genomic Analysis, Genesky Biotechnologies Inc. (Shanghai, China), under −20°C preservation and dry ice conditions. High-fidelity PCR was utilized to amplify the bacterial 16S rRNA hypervariable region 4 (V4) with the specific primer with a barcode. High-throughput sequencing was performed on the Illumina MiSeq platform with the 2 × 250-bp paired-end method after the library was quantified, mixed, and quality checked. The raw data files were analyzed with the Quantitative Insights into Microbial Ecology (QIIME) software pipeline. All the results were based on sequenced reads and operational taxonomic units (OTUs) clustered into representative groups and assigned to taxonomy using VSEARCH 2.7.1 at 97% similarity. Each representative sequence of OTUs was subjected to taxonomy analysis based on the SILVA bacterial 16S rRNA database. The alpha diversity (Chao1, PD_whole_tree, and Shannon) and beta diversity [principal component analysis (PCA) and non-metric multidimensional scaling (NMDS) analysis] were analyzed to identify the species diversity and composition of the microbiota. The relative abundance of bacteria was expressed as percentage.

### Measurement of Short-Chain Fatty Acids

Short-chain fatty acids (SCFAs), such as acetic acid, propionate, butyrate, isobutyrate, and *n*-valeric and *i*-valeric acids, were extracted from mouse feces and detected using high-performance liquid chromatography–mass spectrometry (HPLC/MS), with calculation of the standard curve. Prior to the analysis, the appropriate amount of cecal content or feces was homogenized with five times volume of distilled water by vortexing and then centrifuged at 10,000 × *g* for 5 min; the resulting supernatant was mixed with an equal volume of 2-ethylbutyric acid (internal standard).

### Statistical Analysis

Data are expressed as the mean ± standard deviation(SD). Significance of the difference among groups was assessed with one-way analysis of variance (ANOVA) using GraphPad Prism 8.0 software (GraphPad Software, Inc., La Jolla, CA, USA). The results were considered significant when *p* < 0.05.

## Results

### *S. cerevisiae* Reconstruction and Lactic Acid Expression Verification

**Figure 1:**
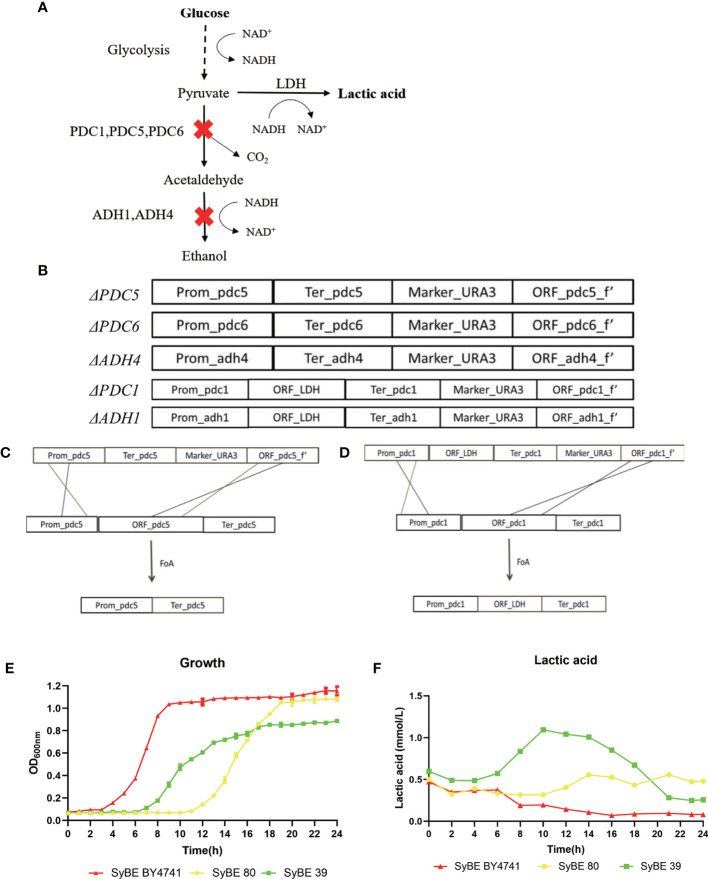
Engineering strategy to improve lactic acid prodution in *Saccharomyces cerevisiae*. **(A)** Schematic diagram of engineering *S. cerevisiae*. **(B**–**D)** Processes to knock out the *PDC5* and *PDC1* genes. **(E)** Growth curves of the three strains SyBE BY4741, SyBE 80, and SyBE 39. **(F)** Lactic acid production of the strains SyBE BY4741, SyBE 80, and SyBE 39.

*S. cerevisiae* BY4741 was used as the cellular chassis. Briefly, we firstly knocked out the genes *PDC1*, *PDC5*, *PDC6*, *ADH1*, and *ADH4* and obtained the strain SyBE_Sc01020080 marked as SyBE 80 (**Figure 1A**). We performed several steps to obtain the *PDC1* gene-deleting module ΔPDC1. Firstly, four simultaneous PCR reactions were performed to obtain the fragments of the promoter, terminator, *URA3* gene, and part of the open reading frames (ORFs) of the gene *PDC1*, which were amplified using the primers P_p5_F/P_p5_R, T_p5_F/T_p5_R, U_p5_F/U_p5_R, and O_p5_F/O_p5_R, respectively (**Figure 1B**). Then, the four fragments were used as the templates and were overlapped in the second PCR reaction to obtain full-length ΔPDC1. After gel purification and digestion, the full-length ΔPDC1 products were then transformed into *S. cerevisiae* BY4741 to obtain the strain BY4741/ΔPDC1. The principle of the gene knockout is shown in **Figures 1B–D**. After being cultivated in an agar plate with FoA, the gene *URA3* was deleted and ΔPDC1 was acquired. Following the above procedures, the other deleting modules, such as ΔPDC5, ΔPDC6, ΔADH1, and ΔADH4, were subsequently acquired. Then, one exogenous gene, *LDH*, was inserted at the *PDC1* and *ADH1* knockout positions at the same time in order to obtain the strain SyBE_Sc01020039, marked as SyBE 39. To evalutate the production of lactic acid, SyBE BY4741, SyBE 80, and SyBE 39 were cultured in liquid YPD medium containing 2% glucose. The growth curves of the three strains are shown in **Figure 1E**. SyBE BY4741 can grow with glucose as the principal energy source. SyBE BY4741 reached the stationary phase after 8 h of incubation, while SyBE 80 and SyBE 39 reached the stationary phase after 18 h. In addition, the lactic acid concentrations reached 0.196 and 0.318 mmol/L in SyBE BY4741 and SyBE 80 after 10 h of incubation, respectively (**Figure 1F**), while SyBE 39 produced 1.094 mmol/L after 10 h, which was approximately 5.58 times higher than that of SyBE Y4741.

### Lactic Acid-Producing *S. cerevisiae* Alleviated the Clinical Symptoms of DSS-Induced Colitis

**Figure 2:**
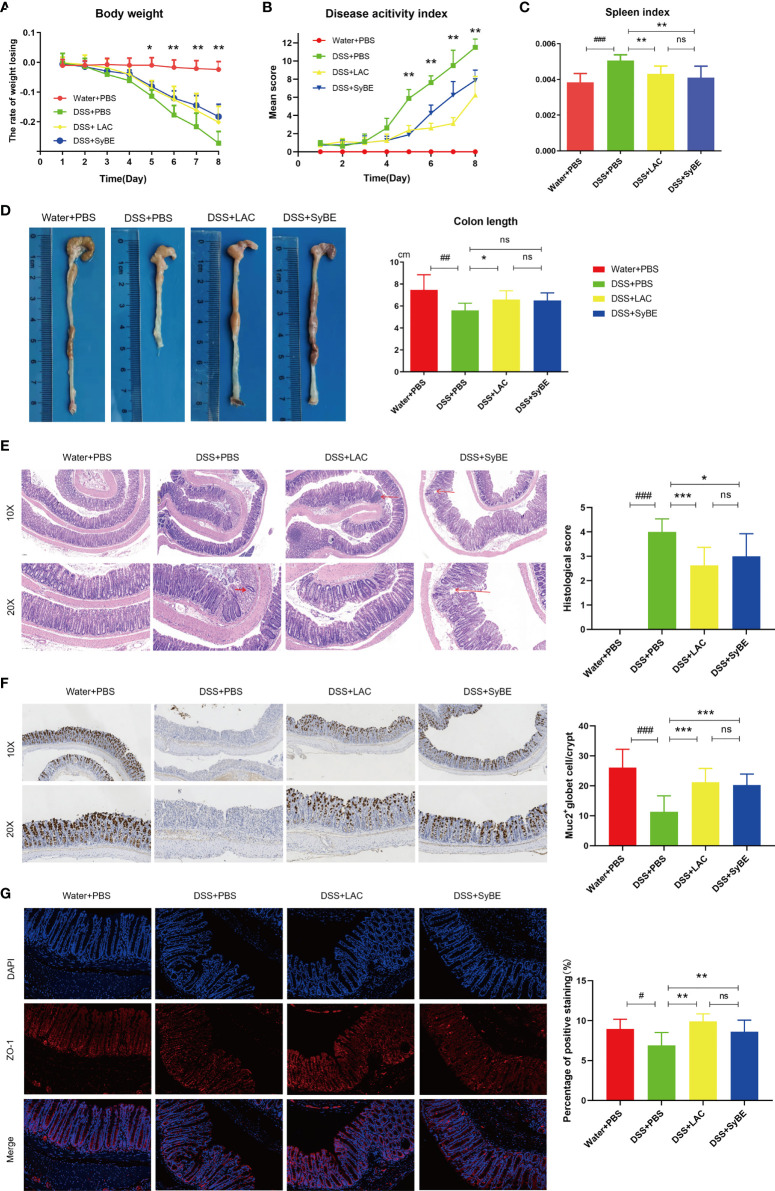
Effects of lactic acid and lactic acid-producing *Saccharomyces cerevisiae* on dextran sulfate sodium (DSS)-induced mouse colitis. **(A)** The body weight of mice was evaluated in the experiment. **(B)** The disease activity index was measured daily. **(C, D)** The spleen index and colon length were measured. **(E)** The distal colon was stained with hematoxylin and eosin to determine the degree of inflammation. **(F)** Immunohistochemistry stain against MUC2. **(G)** Immunofluorescent staining against ZO-1 in colon. Statistical analysis was performed using one-way ANOVA, with comparison with the DSS group mice. The results are based on eight mice per group. *Error bars* represent standard errors. Values represent the mean ± SD of the mean. ^#^ *p* < 0.05, ^##^ *p* < 0.01, ^###^ *p* < 0.001 *vs.* the Water+PBS group; *p < 0.05; **p < 0.01; ***p < 0.001 *vs.* the DSS+PBS group. ns, no significance.

DSS-induced colitis is one of the most prevalently used *in vivo* models that mimics the development of UC, presenting increased permeability of the mucus layer, infiltration of leukocytes such as neutrophils, monocytes, and macrophages, and secretion of inflammatory cytokines, resulting in epithelial damage. The effect of oral administration of engineered SyBE was assessed in DSS-induced acute colitis model mice. Compared with the Water+PBS group, the weight of the mice treated with DSS decreased over time. In addition, the DSS-induced weight loss was effectively alleviated in the DSS+LAC and DSS+SyBE groups, suggesting that lactic acid treatments remarkably prevented the weight loss caused by DSS (**Figure 2A**). In addition, the DAI results conformed to the results from the weight change (**Figure 2B**). DSS treatment also shortened the colon length on the last day (**Figure 2D**). Colon shortening was positively associated with the colonic inflammation and edema of DSS-induced colitis. Moreover, the spleen index (spleen/body weight) reflected the severity of colitis. Treatment with lactic acid and SyBE significantly extended the length of the colon and reduced the spleen index compared to that of the control group (**Figures 2C, D** **)**. Thus, SyBE treatment significantly ameliorated the disease symptoms of DSS colitis mice. The beneficial effects mediated by lactic acid and SyBE as a preventive schedule were also confirmed by the H&E-stained histological sections and the histopathology scores. The colon of DSS-fed mice showed severe acute colitis, manifested by mucosal structure disorder, inflammatory cell infiltration, crypt loss, ulcers, and epithelial cell necrosis (**Figure 2E**). In contrast, both groups with lactic acid and SyBE treatments were able to protect the colonic mucosa structure and improve the severity of inflammation (*p* < 0.05), resulting in improved colon pathological characteristics and significantly lower histopathological scores (**Figure 2E**). It is worth noting that, although the effect of lactic acid was very similar to that of SyBE, there was a slight inflammation in the LAC+DSS group.

### Lactic Acid-Producing *S. cerevisiae* Restores Intestinal Barrier Function in DSS-Induced Colitis

The intestinal epithelial barrier is the first line of defense of the host to protect from exogenous antigen, which is related to intestinal inflammation. The colonic tight junction proteins ZO-1 and mucin (MUC-2) were localized in the epithelial cell membrane of healthy intestines, but largely depleted in colitis mice. The expression of MUC-2 was increased in the DSS+LAC and DSS+SyBE groups, in contrast to that of the DSS group (**Figure 2F**). Lactic acid and SyBE significantly restored the continuous distribution of ZO-1 throughout the colonic mucosa (**Figure 2G**). In general, both lactic acid and SyBE upregulated the expressions of the tight junction proteins and increased the number of goblet cells in DSS-fed mice, which was similar to that of the control mice.

### Lactic Acid-Producing *S. cerevisiae* Modulated the Immune Response in DSS-Induced Colitis

**Figure 3:**
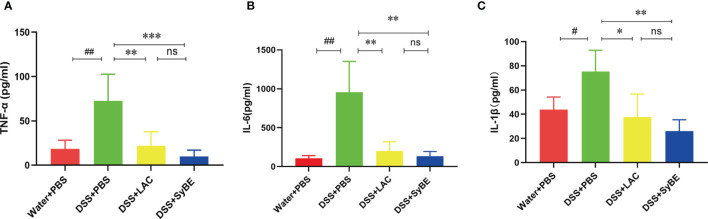
Effect of lactic acid and lactic acid-producing *Saccharomyces cerevisiae* on inflammatory cytokines in the serum of mice. The serum was measured using the LEGENDplex™ mouse inflammatory cytokine panel. **(A**–**C)** TNF-α, IL-6, and IL-1β were measured. Statistical analysis was performed using unpaired *t*-test. *Error bars* represent standard errors. ^#^ *p* < 0.05; ^##^ *p* < 0.01 *vs.* the Water+PBS group; *p < 0.05; **p < 0.01; ***p < 0.001 *vs.* the DSS+PBS group. ns, no significance.

A previous study indicated that lactic acid downregulated the expressions of TNF-α and IL-1β (26). To further confirm the systemic anti-inflammatory properties of SyBE, we analyzed the levels of the pro- and anti-inflammatory mediators in serum. The levels of TNF-α and IL-1β were greater in the DSS group than those in the controls. SyBE treatment similarly reduced the levels of the pro-inflammatory TNF-α, IL-6, and IL-1β compared with those of the DSS group (*p* < 0.05) (**Figures 3A–C**). However, there were no significant differences in the levels of IL-10, IL-12p70, and IFN-γ (data not shown). These results showed that SyBE displayed intestinal anti-inflammatory activity in DSS-induced colitis.

### Lactic Acid-Producing *S. cerevisiae* Inhibited the Polarization of M1 Macrophages *In Vivo* and *In Vitro via* MCT1

**Figure 4:**
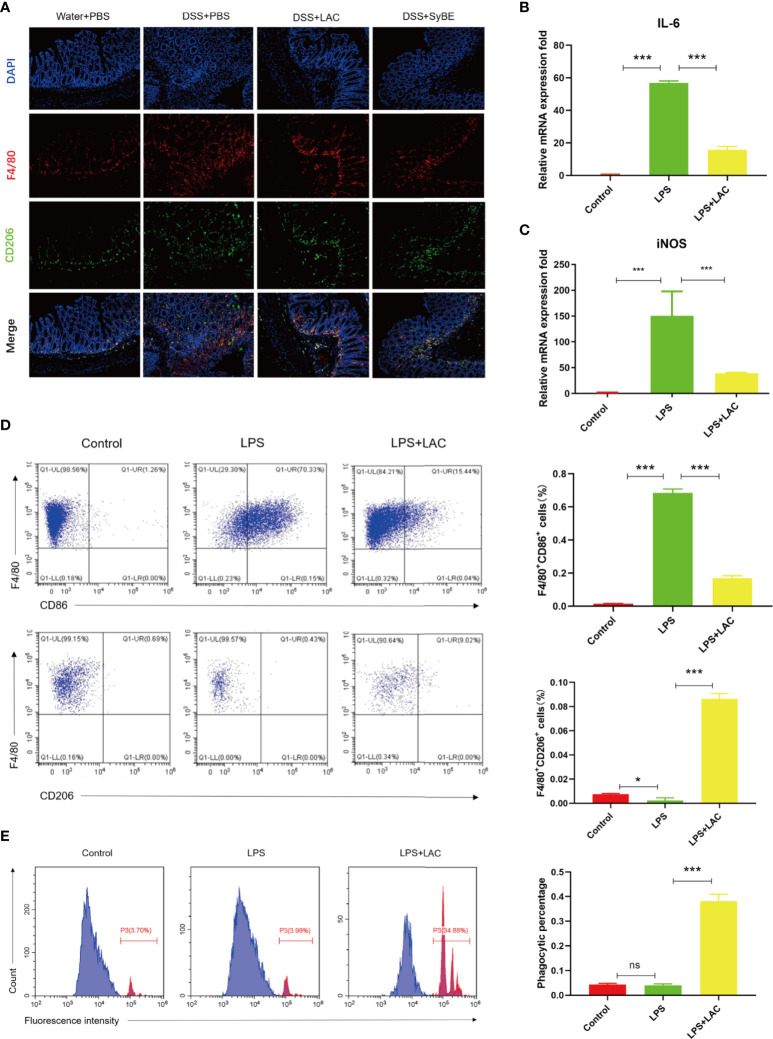
Lactic acid and lactic acid-producing *Saccharomyces cerevisiae* improved macrophage polarization and phagocytosis. **(A)** Lactic acid and SyBE enhanced M2 polarization in the colon of colitis mice. **(B**, **C)** Lactic acid inhibited the expressions of IL-6 and iNOS in bone marrow-derived macrophages (BMDMs) upon stimulation with lipopolysaccharide (LPS). **(D)** Lactic acid inhibited the expression of CD86 and promoted the expression of CD206 in BMDMs. **(E)** Lactic acid promoted the phagocytosis of macrophages. *p < 0.05; ***p < 0.001 *vs.* the LPS group. ns, no significance.

Activated macrophage infiltration plays an important role in the occurrence and development of colitis. M1 and M2 are the major subtypes of macrophages. In this study, F4/80 and CD206 were detected in colonic sections to examine the state of macrophage accumulation and polarization. The results showed that there were more F4/80-labeled macrophages in the DSS group, suggesting an increase in the number of mononuclear macrophages that reside and recruit in the colon (**Figure 4A**). However, there was fewer F4/80-labeled macrophages and much more CD206-labeled M2 macrophage in the DSS+LAC and DSS+SyBE groups, but with no significance between the two groups.

To investigate whether lactic acid has an effect on macrophage polarization, the percentage of CD86^+^ cells and the ratio of CD86^+^ to CD206^+^ cells were examined by flow cytometry analysis on BMDMs. The results showed that the number of M1 macrophages increased with LPS stimulation (**Figure 4D**). Lactic acid treatment decreased the number of CD86^+^ cells and increased the percentage of CD206-labeled M2 macrophages. Furthermore, lactic acid decreased the mRNA expressions of the iNOS and IL-6 of the M1-like phenotype in LPS-stimulated cells, which was consistent with the results of flow cytometry (**Figures 4B, C**). Subsequently, the phagocytic capacity of macrophages was evaluated by measuring the phagocytosis of the carboxylate-modified polystyrene latex beads into cells with flow cytometry. The results showed that LPS enhanced the phagocytic capability of macrophages compared with the control group, while lactic acid significantly decreased the mean fluorescence intensity with LPS stimulation (**Figure 4E**).

Overall, lactic acid mediated the shift from the M1- to the M2-like profile and the phagocytosis of the macrophages.

### Lactic Acid-Producing *S. cerevisiae* Inhibited NLRP3 Inflammasome Activation *In Vivo* and *In Vitro*

As lactic acid treatment significantly decreased the level of the pro-inflammatory cytokine IL-1β, which is an inflammatory cytokine released by macrophage pyroptosis in colitis, in DSS-fed mice, we decided to explore the effect and the mechanism of lactic acid on macrophage pyroptosis.

**Figure 5:**
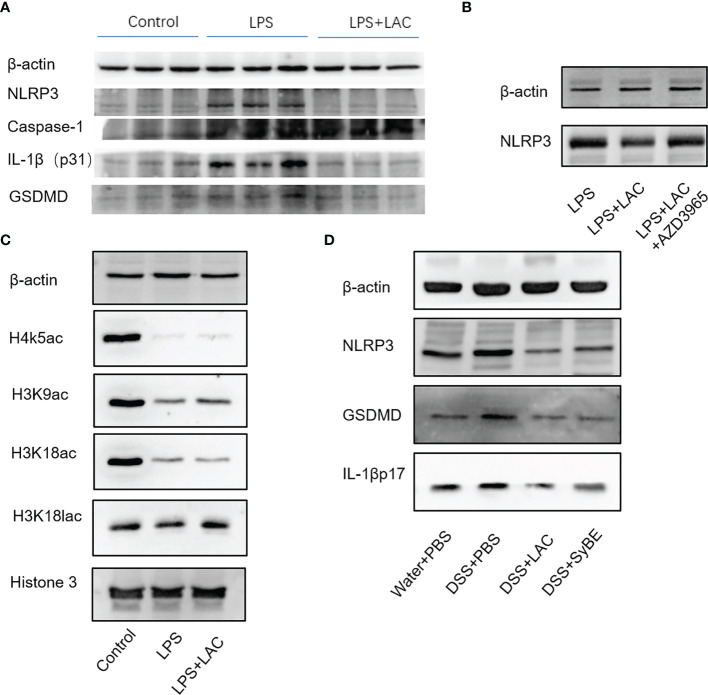
Lactic-acid and *Saccharomyces cerevisiae* inhibited NLRP3 inflammasome activation *in vivo* and *in vitro*. **(A)** Lactic acid downregulated the expressions of NLRP3, caspase-1, IL-1βp31, and gasdermin D (GSDMD) in bone marrow-derived macrophages (BMDMs). **(B)** With AZD3965 pretreatment, lactic acid did not inhibit the expression of NLRP3 upon stimulation with LPS in BMDMs. **(C)** Lactic acid enhanced the expressions of H3K9ac and H3K18lac in BMDMs. **(D)** Lactic acid and SyBE inhibited the expressions of NLRP3, GSDMD, and IL-1βp17 in dextran sulfate sodium (DSS)-induced colitis.

The inflammasome is an important part of the inflammatory response. The functional NLRP3 inflammasome is formed by a variety of secondary signals such as potassium efflux, reactive oxygen species (ROS) production, lysosome rupture, and mitochondrial stress. The inflammasome is a pyroptosis platform composed of multiple proteins, including the inflammasome sensor NLRP3, the adaptor ASC, and the effector molecule caspase-1. Caspase-1 self-cleavage activates and cleaves pro-IL-1β and pro-IL-18 to form active IL-1β and IL-18, respectively. Gasdermin D (GSDMD) forms pores in the host cell membrane to release IL-1β outside the cell. Therefore, we estimated the levels of NLRP3, caspase-1, ASC, and GSDMD under treatment of LPS and/or lactic-acid (7). Upon stimulation with LPS, the expressions of NLRP3, caspase-1 p45, and IL-1β p17 increased. When primed with lactic acid, the expressions of the NLRP3 inflammasome, IL-1β p17, caspase-1 p45, ASC, and GSDMD were diminished (**Figure 5A**). On the other hand, we also evaluated the activation of the NLRP3 inflammasome in mice. Compared with the control group, DSS significantly increased NLRP3, caspase-1 p45, GSDMD, and IL-1β p31/17 in colonic tissues. Treatment with lactic acid and SyBE significantly suppressed the macrophage pyroptosis to maintain tissue immune homeostasis (**Figure 5D**). Moreover, activation of the NLRP3 inflammasome also mediated M1 macrophage polarization. Our results confirmed that lactic acid-producing *S. cerevisiae* not only suppressed M1 macrophage polarization but also inhibited NLRP3 inflammasome, which indicated that lactic acid-producing *S. cerevisiae* may exert this synergistic effect in colitis.

Lactic acid transport is mainly carried out by monocarboxylate transporters (MCTs), which belong to the solute carrier 16A family (SLC16A). AZD3965 is a specific MCT1 transporter inhibitor that can inhibit the transportation of lactic acid in macrophages. With AZD-3965 pretreatment, the activation of NLRP3 and GSDMD could not be decreased by lactic acid treatment with LPS stimulation (**Figure 5B**).

We also assessed the effect of lactic acid on the regulation of the transcriptional levels of the macrophages involved in the pathogenesis of colitis. Lactic acid, used as a therapeutic, was able to significantly increase histone H3 K9 acetylation and histone H3 K18 lactylation in BMDMs (**Figure 5C**). Therefore, we speculated that some genes or proteins regulated by acH3K9 and laH3K18 may be involved in the inhibitory effect of lactic acid.

### Lactic Acid-Producing *S. cerevisiae* Shaped the Intestinal Microbiota After Oral Gavage

**Figure 6:**
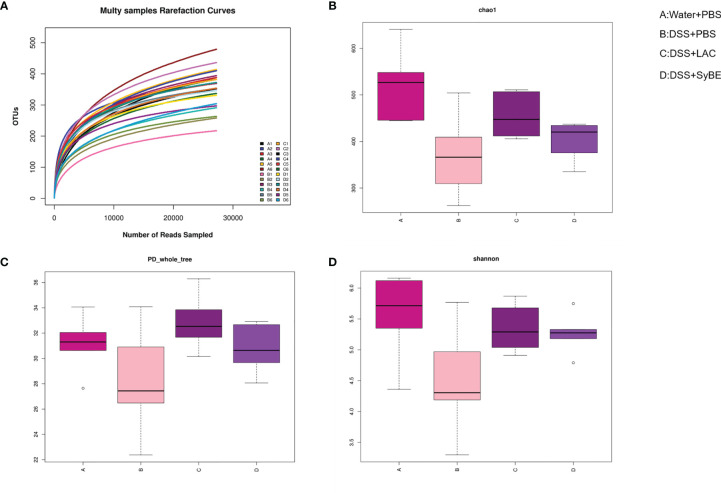
Alpha diversity of the gut microbiota and rarefaction curves. **(A)** Rarefaction curves. **(B)** Chao1 index of all samples. **(C)** PD_whole_tree index of all samples. **(D)** Shannon index of all samples based on the operational taxonomic unit (OTU) level. **(A)** Water+PBS; **(B)** DSS+PBS; **(C)** DSS+LAC; **(D)** DSS+SyBE (*n* = 6).

Gut dysbiosis contributes to the development of UC. Having explored the immunomodulatory effect of lactic acid-producing *S. cerevisiae*, we expected that it could modulate the gut microbiota in colitis. 16S rRNA sequencing of the V3–V4 regions was performed to evaluate alterations in the gut microbiota. The rarefaction curve of the Sobs index denotes that the sequencing depth was enough and reasonable and that the sequencing data were credible (**Figure 6A**). The α-diversity values reflect the richness and relative diversity of species in a microbial community. DSS treatment significantly reduced the community richness (Chao1) and community diversity (PD_whole_tree and Shannon) (**Figures 6B–D**), while lactic acid and SyBE supplementation significantly improved these indexes. All indexes clearly indicated that lactic acid and *S. cerevisiae* increased the gut microbiota diversity of DSS-induced colitis mice.

**Figure 7:**
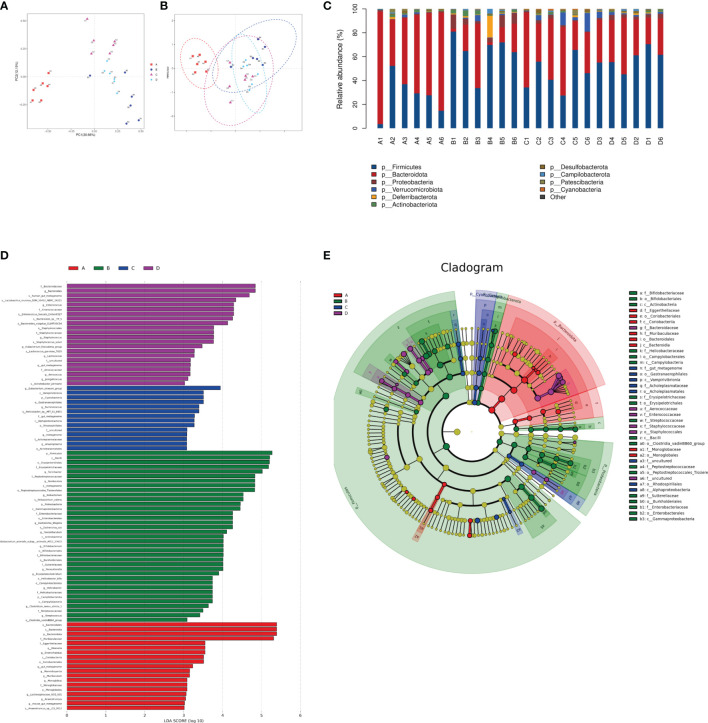
Effect of lactic acid and *Saccharomyces cerevisiae* on the species composition of the gut microbiota. **(A)** Principal component analysis (PCA). **(B)** Non-metric multidimensional scaling (NMDS) analysis. **(C)** Relative abundance at the phylum level. **(D)** Differentially enriched intestinal microbiota in all groups at the genus level by linear discriminant analysis (LDA). An LDA score higher than 3 represents a higher abundance in the group than that in other groups. **(E)** Cladogram based on linear discriminant analysis effect size (LEfSe) analysis. **(A)** Water+PBS; **(B)** DSS+PBS; **(C)** DSS+LAC; **(D)** DSS+SyBE (*n* = 6).

The species composition is displayed in **Figure 7**. PCA and NMDS analysis showed that DSS significantly changed the gut microbial structure compared to the control group (**Figures 7A, B** **)**. At the phylum level, the ratio of Firmicutes to Bacteroides increased in mice in the DSS group, while it decreased in mice in the DSS+LAC and DSS+SyBE groups. Both lactic acid and *S. cerevisiae* appeared to regulate the abnormal gut microbiota in DSS-induced UC mice (**Figure 7C**). At the genus level, the relative abundance of *Parasutterella*, *Erysipelatoclostridium*, *Streptococcus*, *Bacteroides*, *Escherichia*–*Shigella*, *Faecalibaculum*, *Coprobacillus*, and *Prevotellaceae_UCG-001* significantly increased in the DSS+PBS group compared to that in the Water+PBS group, but decreased upon lactic acid and SyBE treatment. The relative abundance of *Oscillibacter*, *Bacteroides*, *Roseburia*, *Lactobacillus*, *Muribaculum*, *Rikenella*, and *Faecalibacterium* decreased in the DSS+PBS group, whereas lactic acid and SyBE treatment increased the relative abundance of these species. Linear discriminant analysis effect size (LEfSe) analysis discovered the dominant microbiota and biomarkers at the family and genus levels for each group, with a threshold of 3 (**Figures 7D, E** **)**. At the genus level, the dominant microbiota in the Water+PBS group were *Rikenella*, *Enterorhabdus*, *Marvinbryantia*, *Muribaculum*, and *Monoglobus*. The dominant microbiota in the DSS+PBS group were *Turicibacter*, *Romboutsia*, *Ileibacterium*, *Escherichia*_*Shigella*, *Faecalibaculum*, *Bifidobacterium*, *Parasutterella*, *Erysipelatoclostridium*, *Helicobacter*, *Clostridium*, *Streptococcus*, and Lachnospiraceae. The dominant microbiota in DSS+LAC group were *Eubacterium_siraeum*, *Ruminococcus*, and *Anaeroplasma*. The dominant microbiota in the DSS+SyBE group were *Bacteroides*, *Enterococcus*, *Staphylococcus*, *Eubacterium_fissicatena*, *Lactococcus*, *Aerococcus*, and *Jeotgalicoccus*. Moreover, supplementing SyBE increased the abundance of certain Lachnospiraceae, such as *Lachnospiraceae_bacterium_COE1* and *Lachnospiraceae_bacterium_28-4*, which can metabolize lactic acid to produce butyrate. In summary, there were great differences among the DSS+PBS, DSS+LAC, and DSS+SyBE groups. These results indicated that lactic acid-producing *S. cerevisiae* modulated the structure of the gut microbiota in DSS-induced colitis.

### Supplemental Lactic Acid Increased the Content of SCFAs in Mouse Feces

**Figure 8:**
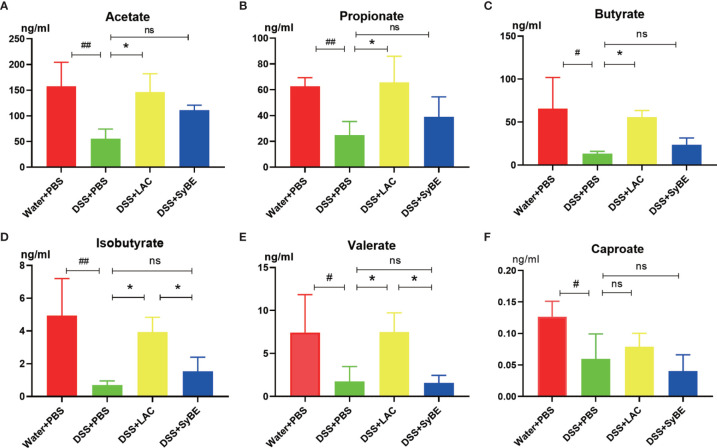
Short-chain fatty acids in colitis mice significantly showed lower levels of acetate **(A)**, propionate **(B)**, butyrate **(C)**, isobutyrate **(D)**, valerate **(E)**, and caproate **(F)**. ^#^ *p* < 0.05; ^##^ *p* < 0.01 *vs.* the Water+PBS group; *p < 0.05 *vs.* the DSS+PBS group (*n* = 6). ns, no significance.

SCFAs, such as acetic acid, propionic acid, and butyric acid, are the metabolites of the fermentation of intestinal dietary fiber bacteria, which have important immunomodulatory functions in the gut. As lactic acid supplementing SyBE altered the microbiota in colitis mice, we wondered whether it could regulate the contents of SCFAs in colitis mice. Herein, we detected the levels of SCFAs in the feces of mice fed with lactic acid and SyBE using HPLC/MS (**Figure 8**). In the DSS+PBS group, the contents of all SCFAs in mouse feces were significantly reduced compared to those in the Water+PBS group. Supplementation with lactic acid increased the contents of acetate, propionate, butyrate, isobutyrate, and valerate (*p* < 0.05), while SyBE treatment did not significantly change the contents of SCFAs. These results showed that the decrease of SCFAs caused by DSS could be recovered by supplementing with lactic acid.

## Discussion

UC is a complex disease involving the host, microorganisms, and environmental factors. DSS-induced experimental colitis is a well-established model that can be used to understand the pathogenesis of UC. We conducted the first study on the modification of the *S. cerevisiae* strain to enhance its lactic acid production and applied it for UC treatment. Our results demonstrated that the engineered *S. cerevisiae* could alleviate DSS-induced colitis, as evidenced by relieving the weight loss and bleeding and the DAI. Lactic acid and SyBE also significantly reduced the contents of pro-inflammatory cytokines, such as TNF-α, IL-6, and IL-1β, in serum, that promote the development of colitis (27). This study also observed the neutrophil infiltration in the DSS group, which was decreased by lactic acid treatment. Moreover, the intestinal epithelium barrier is the first physical line of defense to prevent the invasion of pathogens in the gut (28). The intestinal epithelium was damaged in the DSS group, presenting as mucosal structure disorder, inflammatory cell infiltration, crypt loss, ulcers, and epithelial cell necrosis. Lactic acid and SyBE improved the histological damage with lower histological scores. Moreover, a reduction of MUC2 contributes to bacterial colonization and inflammation. DSS treatment significantly reduced the expression of the MUC2 protein in colonic crypts, but lactic acid treatment restored this change. In addition, the tight junction protein ZO-1 usually represents the integrity of the intestinal barrier. We found that lactic acid and the lactic acid-producing *S. cerevisiae* showed protective effects.

Lactic acid exerts various bioactive properties to regulate innate immunity and reduce the pro-inflammatory cytokines (29). Moreover, lactate abrogates the TLR- and IL-1β-dependent activation of intestinal epithelial cells in starvation-refed mice (30). Moreover, luminal lactate also stimulated enterocyte proliferation, which contributed to maintaining the intestinal barrier (31). Furthermore, oral administration of lactate enhanced the dendrite protrusion of CX3CR1^+^ cells in the small intestine (32). However, the underlying mechanism of how lactic acid exerts its anti-inflammatory activities in colitis remains to be explored.

Increasing evidence suggested that targeting macrophage polarization benefits UC treatment (33–35). The different polarization states of macrophages show diverse immunological responses. However, it is unknown whether lactic acid regulates macrophage polarization in UC. The immune metabolism in macrophages is closely related to their activation state and function. Metabolic adaptation in an inflammatory environment is the key to the plasticity of macrophages. M1 and M2 macrophages have unique metabolic programs. Our data also showed that lactic acid promoted macrophage polarization toward the M2 phenotype. There are many subtypes of M2 macrophages, such as M2a, M2b, and M2c, which exert different functions. M2a macrophages are intrinsically related to wound healing and anti-inflammatory (after exposure to IL-4 or IL-13), M2b macrophages have been described as either pro- or anti-inflammatory (immune response to IL-1β or LPS), and M2c macrophages exert tissue remodeling (immune response to IL-10). Lastly, latex beads were used to evaluate the phagocytosis. It was found that lactic acid significantly increased M2 macrophage polarization and promoted the phagocytic ability.

**Figure 9:**
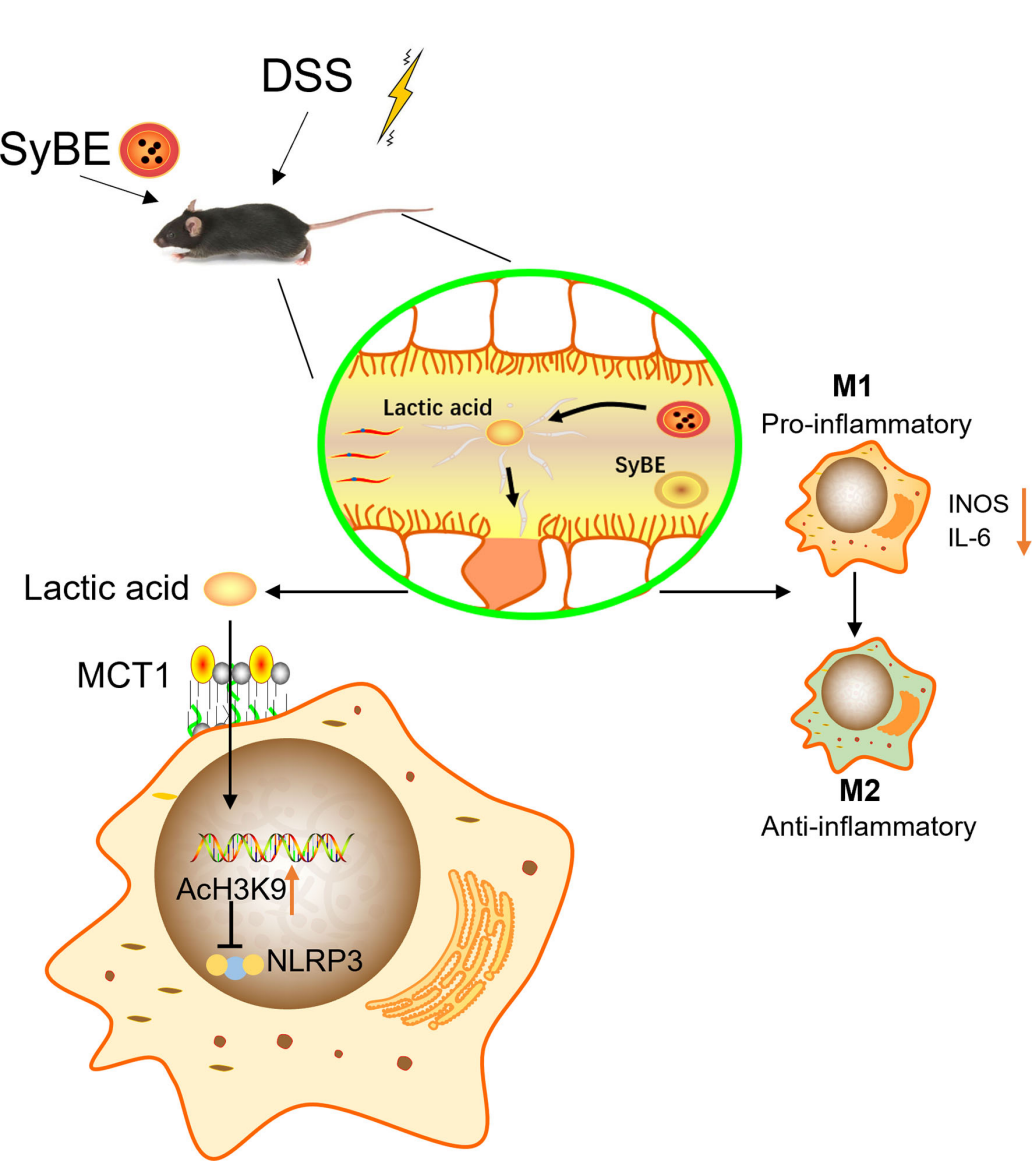
Lactic Acid-Producing probiotic *Saccharomyces cerevisiae* (SyBE) attenuates DSS-induced mice colitis via suppressing macrophage pyroptosis and modulating gut microbiota. Additionally, SyBE modulated the macrophage polarization state in colitis. SyBE: *Saccharomyces cerevisiae*. MCT1: monocarboxylic acid transporter 1. DSS, dextran sulphate sodium.

Pyroptosis is an inflammatory programmed cell death that is important in host defense by linking innate and adaptive immunity (8). However, it is still controversial whether it is beneficial or pathogenic. Some studies have shown that NLRP3^−/−^ deficiency resulted in decreased intestinal inflammation (36, 37), while other studies have suggested that abnormal activation of the NLRP3 inflammasome released a lot of pro-inflammatory cytokines, such as IL-1β and IL-18, which disrupted the intestinal barrier (38, 39). We found that the NLRP3 inflammasome was overactivated in mice in the DSS group, and lactic acid treatment suppressed the expressions of NLRP3, IL-1β, and the pyroptosis executive protein GSDMD. A previous study showed that lactate exerts anti-inflammatory activities *via* GPR81 in DSS-induced colitis (16). When we blocked the lactic acid transporter MCT1, the expression of NLRP3 decreased, indicating that lactic acid exerted an anti-inflammatory function *via* MCT1 in macrophages rather than binding to GPR81. What is more is that certain microorganisms in the intestine can use lactic acid and acetic acid to synthesize butyric acid, which can prevent the accumulation of lactic acid and stabilize the intestinal environment. To further validate the underlying mechanism of lactic acid, we examined the epigenetic change. Our results showed that LPS decreased H3K9 acetylation and H3K18 lactylation, but lactic acid reduced this inhibitory effect, indicating that some genes or proteins regulated by H3K9 acetylation and H3K18 lactylation were involved, which needs to be further explored. Our results provide new insights into the role of the NLRP3 inflammasome in pathophysiology and suggest that NLRP3 is a potential therapeutic target to inhibit macrophage activation and prevent IBD. Furthermore, the activation of the NLRP3 inflammasome may induce the polarization of M1 macrophages and increase the secretion of pro-inflammatory cytokines (40). Inhibiting excessive NLRP3 activation can alleviate the inflammatory response in the colitis. Herein, we found that lactic acid-producing *S. cerevisiae* not only suppressed M1 macrophage polarization but also inhibited the NLRP3 inflammasome *in vivo* and *in vitro*, indicating that lactic acid-producing *S. cerevisiae* may exert this synergistic effect for protection from colitis (**Figure 9**).

Increasing evidence suggests that dysbiosis in the gut promotes the pathogenesis of UC. DSS-treated mice also exhibited dysbiosis, including reduced microbial diversity, reduced abundance of probiotics, and elevation of pathogenic bacteria (41, 42). Herein, lactic acid and SyBE significantly improved the alpha diversity and changed the species composition in DSS colitis mice. At the phylum level, lactic acid treatment reversed the DSS-induced changes in the relative abundance of bacteria. DSS treatment largely increased the portion of potentially harmful bacteria such as *Parasutterella*, *Helicobacter*, *Streptococcus*, and *Escherichia–Shigella* and increased the portion of beneficial bacteria such as *Oscillibacter*, *Roseburia*, and *Muribaculum*. Beta diversity indicates the change of the microbial community composition. PCA and NMDS analysis showed that the microbial community structure was significantly different between the DSS group and the control group. Both lactic acid and SyBE changed the composition in DSS colitis mice. However, lactate accumulation is associated with dysbiosis, for example, in severe colitis, which may result in part from a lack of lactate-utilizing bacteria (43). SCFAs are important microbial metabolites and have diverse beneficial effects on host immunity and metabolism (44, 45). Lactic acid can be metabolized to SCFAs in the gut (46, 47). We found that supplementation with lactic acid increased the abundance of lactic acid-utilizing bacteria in the DSS+LAC and DSS+SyBE groups, such as Lachnospiraceae, which may convert lactic acid into propionate and butyrate (48, 49). Meanwhile, the abundance of some SCFA-producing bacteria, such as *Oscillibacter*, *Rikenella*, and *Faecalibacterium*, were reduced in DSS-treated mice, while it increased in the DSS+LAC and DSS+SyBE groups. We detected the levels of SCFAs and found that supplementation with lactic acid increased the levels of acetate, propionate, and butyrate in the feces of colitis mice.

Fungal communities also exist in the intestinal tract, which affect intestinal health and diseases by inhibiting the growth of potential pathogenic bacteria, promoting immunoregulation and regulating host metabolism (50). *S. cerevisiae* is a unicellular facultative anaerobic fungus and an important part of the normal fungal microbiota. Previous studies have shown that *S. cerevisiae* promoted purine metabolism in mice, leading to increased uric acid levels, and had a direct pro-inflammatory effect and increased the permeability of the intestinal barrier (51). However, the causal relationship between specific fungi and diseases remains to be clarified. *S. boulardii* is a subtype of *S. cerevisiae* and has been reported to have anti-inflammatory effects in colitis (52, 53). In contrast to that in healthy people, the proportion of *S. cerevisiae* in the gut microbiota is decreased in IBD patients and is extremely deficient in patients with colorectal cancer (54). In addition, *S. cerevisiae* CNCMI-3856 has been proven to alleviate adherent-invasive *E. coli* (AIEC)-induced colitis by inhibiting the adhesion of AIEC to intestinal epithelial cells and recovering the intestinal barrier (55). Furthermore, *S. cerevisiae* mono-colonization improved the mortality and colonic shortening in commensal bacteria-depleted mice. Additionally, it also reduced the susceptibility to intranasal influenza A virus infection, which was mediated by mannans (56), as CD206 is a receptor of mannose existing in the membrane and intracellular parts in macrophages (57). In this study, oral administration of lactic acid-producing *S. cerevisiae* increased the F4/80^+^CD206^+^ macrophages in colitis mice. Lactic acid-producing *S. cerevisiae* may exert protective effects *via* the mannose receptor in macrophages with the synergistic effect of lactic acid. The underlying mechanism needs to be further explored. Moreover, it is speculated that *S. cerevisiae* may be difficult to adapt to the inflammatory environment or may have potential anti-inflammatory effects. This makes it possible to use *S. cerevisiae* as a new treatment, similar to several bacterial treatments currently under development. Lactic acid-producing *S. cerevisiae* not only can regulate mucosal immunity and inhibit colonic inflammation but also has strong acid resistance. *S. cerevisiae* can reach the intestine through low-pH gastric acid, which makes up for the defect of other probiotics that cannot easily pass through the stomach.

## Conclusion

This study aimed to evaluate an engineered lactic acid-producing *S. cerevisiae* with high production, reducing the excessive activation of macrophage pyroptosis and regulating the intestinal microbiota to prevent UC. It is inferred that using lactic acid as a driving factor will help in developing strategies to control the potential consequences of microbiota dysbiosis during intestinal inflammation, such as the deterioration of mucosal inflammation, and provide new ideas and evidence for the use of probiotics in the treatment of UC.

## Data Availability Statement

The original contributions presented in the study are included in the article/supplementary files. Further inquiries can be directed to the corresponding authors.

## Ethics Statement

The animal study was reviewed and approved by the Institutional Review Board (or Ethics Committee) of Radiation Medicine Chinese Academy of Medical Sciences. Written informed consent was obtained from the owners for the participation of their animals in this study.

## Author Contributions

SS, XXu, and LL carried out the studies and drafted the manuscript. XW, XB, LZ, QH, and HL participated in the collection of data. XXi, LW, CL, and XCa performed the statistical analysis and participated in its design. XCh, BW, BL, and JZ helped draft the manuscript. All authors contributed to the article and approved the submitted version.

## Funding

This work was supported by the National Key R&D Program of China (2019YFB1311502 and 2019YFB1311505), the National Natural Science Foundation of China (Grant No. 81970477), the Natural Science Foundation of Tianjin (Grant No. 18JCQNJC80700), Tianjin Science and Technology Innovation Cooperation Project (Grant No. 19PTZWHZ00090), and Tianjin Research Innovation Project for Postgraduate Students (Grant No. 2019YJSS188).

## Conflict of Interest

The authors declare that the research was conducted in the absence of any commercial or financial relationships that could be construed as a potential conflict of interest.

## Publisher’s Note

All claims expressed in this article are solely those of the authors and do not necessarily represent those of their affiliated organizations, or those of the publisher, the editors and the reviewers. Any product that may be evaluated in this article, or claim that may be made by its manufacturer, is not guaranteed or endorsed by the publisher.

## Abbreviations

IBD, inflammatory bowel disease; UC, ulcerative colitis; DSS, dextran sulfate sodium; PRR, pattern recognition receptor; MCT1, monocarboxylic acid transporter 1; LBP, live biotherapeutic product; GSDMD, gasdermin D; SCFAs, short-chain fatty acids; BMDM, bone marrow-derived macrophage.
